# Repeatability and Temporal Consistency of Lower Limb Biomechanical Variables Expressing Interlimb Coordination during the Double-Support Phase in People with and without Stroke Sequelae

**DOI:** 10.3390/s23052526

**Published:** 2023-02-24

**Authors:** Ana G. B. Couto, Mário A. P. Vaz, Liliana Pinho, José Félix, Juliana Moreira, Francisco Pinho, Inês Albuquerque Mesquita, António Mesquita Montes, Carlos Crasto, Andreia S. P. Sousa

**Affiliations:** 1 Department of Physiotherapy, Santa Maria Health School, 4049-024 Porto, Portugal; 2 Centre for Rehabilitation Research (CIR), School of Health of Polytechnic Institute of Porto, 4200-072 Porto, Portugal; 3 Faculty of Engineering, University of Porto, 4200-465 Porto, Portugal; 4 Research Centre and Projects (NIP), Santa Maria Health School, 4049-024 Porto, Portugal; 5 Institute of Mechanical Engineering and Industrial Management, Faculty of Engineering, University of Porto, 4200-465 Porto, Portugal; 6 Porto Biomechanics Laboratory (LABIOMEP), University of Porto, 4200-450 Porto, Portugal; 7 College of Health Sciences—Escola Superior de Saúde do Vale do Ave, Cooperative for Higher, Polytechnic and University Education, 4760-409 Vila Nova de Famalicão, Portugal; 8 Faculty of Sport, University of Porto, 4200-450 Porto, Portugal; 9 Department of Physics, School of Health of Polytechnic Institute of Porto, 4200-072 Porto, Portugal; 10 Department of Medical Sciences, University of Aveiro, 3810-193 Aveiro, Portugal; 11 Department of Physiotherapy, School of Health of Polytechnic Institute of Porto, 4200-072 Porto, Portugal; 12 Human Movement Unit (H2M), Cooperative for Higher, Polytechnic and University Education, 4760-409 Vila Nova de Famalicão, Portugal; 13 Department of Functional Sciences, School of Health of Polytechnic Institute of Porto, 4200-072 Porto, Portugal

**Keywords:** post-stroke, gait, human movement variability, kinematic parameters, kinetic parameters, electromyographic parameters, test–retest reliability

## Abstract

Reliable biomechanical methods to assess interlimb coordination during the double-support phase in post-stroke subjects are needed for assessing movement dysfunction and related variability. The data obtained could provide a significant contribution for designing rehabilitation programs and for their monitorisation. The present study aimed to determine the minimum number of gait cycles needed to obtain adequate values of repeatability and temporal consistency of lower limb kinematic, kinetic, and electromyographic parameters during the double support of walking in people with and without stroke sequelae. Eleven post-stroke and thirteen healthy participants performed 20 gait trials at self-selected speed in two separate moments with an interval between 72 h and 7 days. The joint position, the external mechanical work on the centre of mass, and the surface electromyographic activity of the tibialis anterior, soleus, gastrocnemius medialis, rectus femoris, vastus medialis, biceps femoris, and gluteus maximus muscles were extracted for analysis. Both the contralesional and ipsilesional and dominant and non-dominant limbs of participants with and without stroke sequelae, respectively, were evaluated either in trailing or leading positions. The intraclass correlation coefficient was used for assessing intra-session and inter-session consistency analysis. For most of the kinematic and the kinetic variables studied in each session, two to three trials were required for both groups, limbs, and positions. The electromyographic variables presented higher variability, requiring, therefore, a number of trials ranging from 2 to >10. Globally, the number of trials required inter-session ranged from 1 to >10 for kinematic, from 1 to 9 for kinetic, and 1 to >10 for electromyographic variables. Thus, for the double support analysis, three gait trials were required in order to assess the kinematic and kinetic variables in cross-sectional studies, while for longitudinal studies, a higher number of trials (>10) were required for kinematic, kinetic, and electromyographic variables.

## 1. Introduction

Stroke has been classified as the third leading cause of death and disability in the world [1]. The related sensorial and motor repercussions can cause limitations in activities of daily living and participation restrictions in both professional and social contexts [2]. It has been indicated that gait impairment is a common clinical problem present in more than 80% of stroke survivors [3], with a great impact on functional independence [4]. Therefore, gait recovery is one of the main objectives for post-stroke patients and their rehabilitation [5,6,7].

The main changes in post-stroke gait are mainly expressed in asymmetric lower limb function [6,8,9,10,11,12]. Impairments in lower limb function and related sensorimotor recovery of post-stroke patients [13,14] have been extensively explored and described at a kinematic level (joint position and double-support phase) [15,16,17,18,19,20], as well as in terms of kinetic (ground reaction forces) [17,18,19,20,21,22] and muscle activation (distal and proximal muscles) [17,20,23,24,25,26]. However, the biomechanical parameters should enable the differentiation between restitution and compensation processes as recommended by the *Stroke Recovery and Rehabilitation Roundtable* (SRRR) [14]. Currently, it is assumed that, after stroke, motor ability might occur via restitution (which reflects the process toward “true recovery”) or compensation [27]. In accordance with the World Health Organisation International Classification of Functioning framework, Levin et al. [28] proposed definitions at three different levels: health condition (neuronal), body functions/structure (performance), and activity (functional). In these three areas, motor recovery relates to restoration of function in neural tissue that was initially lost; restoration of ability to perform movement as prior to injury; and accomplishing successful tasks as is typical in non-disabled individuals. In motor compensation, these three areas include the neural tissue acquisition of a new function that was not there before the injury; a new movement performance; and a successful completion of a task using different techniques [28]. Therefore, the biomechanical analysis allows for an accurate and objective assessment by providing objective and quantitative parameters [14]. The recommendation of specific measures and methods for this analysis is challenging, and the same authors, in 2019, published a consensus of recommendations for specific upper limb tasks. In this consensus, one of the orientations that was studied concerns the minimum number of repetitions necessary to obtain a good stability of performance, and 15 repetitions were proposed in order to achieve good repeatability [29]. By contrast, Frykberg et al. [30] observed that it took two to three repetitions to reach good inter-repetition (intra-session) performance stability and at least three inter-session repetitions for upper limb kinematic measurements in people with and without stroke sequelae.

Although there are no recommendations of the SRRR for the lower limb, this entity reported that the motor performance and movement quality could be analysed by coordination measures to establish the (as)symmetry between the contralesional and ipsilesional sides, such as the spatiotemporal parameters, the ground reaction forces and their torques, and the centre of mass displacement [14]. Specifically, in the double-support phase, Couto et al. [31] identified some methodological considerations for the analysis, namely, the double-support time, the external mechanical work performed by the lower limbs in the step-to-step transition, and the muscle activity developed by the lower limbs and the coactivation ratio, considering the functional position and the role of each lower limb in this gait phase. Although these variables can be used to calculate indices and ratios of (as)symmetry, the combination of individual analyses of each lower limb is also recommended [31]. However, according to our knowledge, the minimum number of trials needed to ensure good stability of performance in biomechanical variables reflecting interlimb coordination during double support in post-stroke patients has not been established yet.

The aim of this study was to analyse the minimum number of trials required for adequate values of repeatability and temporal consistency for kinematic variables (double-support time and position of the hip, knee, and ankle joints), kinetic variables (external mechanical work), and electromyographic variables (muscle activity mean values and muscle activation ratios) in the double-support phase in people with and without stroke sequelae.

In order to accomplish this study’s purpose, performance stability over multiple trials of gait was assessed, in two different moments, in people with and without stroke sequelae, regarding the above-mentioned biomechanical variables. The results were analysed through the minimum number of trials necessary to achieve a good-to-excellent intraclass correlation coefficient (ICC) > 0.75 [32].

## 2. Methods

This section includes the methodologies adopted in the study.

### 2.1. Study Design

A quantitative methodology, with a test–retest study design was conducted to analyse the performance stability of the kinematic, kinetic, and electromyographic variables during the double-support phase of gait, in people “with stroke sequelae”, named in the present study as the “stroke” group, and in people “with no history of a stroke and without self-reported disabilities”, named as the “healthy” group. The performance stability over a set of multiple trials was assessed intra-session (repeatability) and inter-session (temporal consistency) and was then analysed. For this purpose, all participants performed 20 gait trials at a self-selected speed in two different moments.

### 2.2. Participants

A group of 11 subjects (3 females and 8 males) with history of a single unilateral ischemic stroke affecting the right (n = 3) and left (n = 8) hemispheres, resulting in a motor control dysfunction of the contralesional lower limb (CONTRA), and group of 13 healthy subjects (4 females and 9 males) participated in the present study (Table 1). Participants were excluded from the healthy group if they had one or more of the following criteria: altered mental state with interference in communication and cooperation [6,25,26]; history or sign of neurological dysfunction [33]; presence of pain that interfered with the performance of walking [34]; history of anatomical deformities, osteoarticular or musculotendinous injury, or lower limb surgery in the last 6 months [33,34]; exposition to medication with interference in the motor performance of the lower limb [25,26]; and practice of moderate (i.e., at least 30 min 5 days a week) or vigorous (i.e., at least 20 min 3 days a week) levels of physical activity [35].

To be included in the stroke group, the participants needed to fulfil the following criteria: diagnosis of ischemic first-ever stroke with at least 6 months of evolution [6,25,26]; involvement of middle cerebral artery territory, at the subcortical level, confirmed by axial computed tomography [6,26]; lower limb sensorimotor impairment [6]; and ability to walk at least 10 m, with close supervision if necessary, but without physical assistance [6,25]. In turn, people who had any of the exclusion criteria mentioned for the healthy group or the presence of a lesion involving the brainstem or cerebellum were excluded [6,25,26].

### 2.3. Instruments

This section covers the instruments used to collect data for the study.

#### 2.3.1. Sample Selection and Characterisation

A questionnaire was used to verify the participants inclusion and exclusion criteria for the participants characterisation regarding age, sex, dominance, and time of evolution in the stroke group. The body mass (kg) and height (m) were assessed through a seca^®^ 760 scale (seca—Medical Scales and Measuring Systems^®^, Birmingham, UK), with a scale of 0, 1 kg, and a seca^®^ 222 stadiometer (seca—Medical Scales and Measuring Systems^®^, Birmingham, UK), with a 1 mm scale.

The physical activity level was assessed trough the Brief Physical Activity Assessment Tool [36]. It is a simple and quick (<5 min) questionnaire which allows the classification of individuals as sufficiently/insufficiently active [37]. Its classification categories showed good construct validity (0.40 ≤ κ ≤ 0.64; sensitivity = 0.75 95%CI: 0.70–0.79, specificity = 0.74 95%CI: 0.71–0.77 [37]) in patients with various health conditions when compared to accelerometry and to other physical activity questionnaires.

The Mini-Mental State Examination scale was used to assess mental status. It is an 11-question measure that tests 5 areas of cognitive function: memory, attention, calculation, language, and praxis, with a maximum score of 30 points [38]. It is considered that a person has cognitive deficit when the score is ≤15 for illiterates; ≤22 for people with 1 to 11 years of schooling; or ≤27 for people with more than 11 years of school [39]. This instrument has been adapted and validated for the Portuguese population [39,40], with a sensitivity between 63% and 73.4% and a specificity between 90 and 96.8% [40].

The Fugl–Meyer Assessment of Sensorimotor Recovery After Stroke was used to assess post-stroke sensorimotor impairment in the adult population in 5 domains: motor function (upper limb and lower limb), sensory function, balance, passive range of movement, and pain [41]. A person is considered as having sensorimotor impairment of the lower limb if a score lower than 34 is obtained in the respective subsection of the FMA [41,42]. The Portuguese version of the scale was adapted and validated for the Portuguese population [43,44] with excellent internal consistency (Cronbach’s α = 0.99) [43].

#### 2.3.2. Kinematic Data

The joint position of the hip, knee, and ankle in the sagittal plane, on the dominant (DOM) and non-dominant (NDOM) sides in the healthy group and of the ipsilesional (IPSI) and contralesional (CONTRA) sides in the stroke group, as well as the time of the double-support phase, were assessed using an optoelectronic system, the Qualisys Motion Capture System (Qualisys AB, Göteborg, Sweden). The spatial position of reflector markers, placed on the participant, were collected using twelve infrared cameras, eight Oqus 500 and four Miqus M3, connected to the Qualisys USB Analog Acquisition interface, at a sampling frequency of 100 Hz.

#### 2.3.3. Kinetic Data

Ground reaction forces (GRF) and respective torques were used to assess the external mechanical work on the centre of mass (WCOM). The kinetic data were collected using two force platforms (FP4060-08 and FP4060-10 models from Bertec Corporation, Columbus, OH, USA), placed in series near the midpoint of the walkway and connected to a Bertec amplifier AM 6300 at a sampling frequency of 1000 Hz. The capture hardware was connected to the Qualisys Motion Capture System analogue board.

#### 2.3.4. Electromyographic Data

Surface electromyography (sEMG) was monitored using the wireless Trigno TM acquisition system (Delsys Inc., Natick, MA, USA) to bilaterally assess the muscle activity of the tibialis anterior (TA), soleus (SOL), gastrocnemius medialis (GasM), rectus femoris (RF), vastus medialis (VM), biceps femoris (BF), and gluteus maximus (GMax). Pre-amplified bipolar differential electrodes (Trigno Avanti Sensor model, Delsys Inc., Natick, MA, USA) with a rectangular configuration of two Ag bars in parallel (inter-electrode distance of 10 mm) and a gain of 1000 were used to collect the surface electromyography (sEMG) signal, with an acquisition frequency of 1000 Hz. The sEMG signal was integrated into the Qualisys Motion Capture System through an analogue board (National Instruments, Austin, TX, USA). EMGworks software (Delsys Inc., Natick, MA, USA) was used to analyse the sEMG signal quality. An Electrode Impedance Checker^®^ (Noraxon, Scottsdale, AZ, USA) was also used to measure the level of skin impedance.

Qualisys Track Manager software (Qualisys AB, Göteborg, Sweden) was used to display and acquire kinematic, kinetic, and electromyographic data, which were analysed using Visual 3D software, v6x64 (C-Motion, Germantown, MD, USA). The above-mentioned outcomes were considered to describe the double-support phase of gait, of both lower limbs, in the leading limb (LEAD) position (initial contact and loading response) and the trailing limb (TRAIL) position (pre-swing) [45].

### 2.4. Procedures

Data collection took place at a biomechanical laboratory, the Rehabilitation Research Centre of Health School of the Polytechnic Institute of Porto, in a controlled environment. To avoid inter-rater error, each researcher was responsible for only one task. Prior to data collection, anthropometric measures, body mass and height, were recorded for each participant. Then, the body mass index (BMI), expressed in kg/m^2^, was calculated, according to the theoretical Formula (1).

(1) $$(\mathrm{BMI}=\frac{\mathrm{weigth}}{{\mathrm{heigth}}^{2}})\;$$

For the collection of kinematic data, 46 reflective markers were placed bilaterally in anatomical references (identified by manual palpation): apex of the head, earlobes; jugular notch; xiphoid apophysis; spinous apophysis of the seventh cervical vertebral; the lateral part of the acromion; anterior iliac spines superior; posterior superior iliac spines; greater trochanters; lateral and medial epicondyles of the femur; anterior tuberosities of the tibia; the head of the fibula; the lateral and medial malleoli; the posterior surface of the calcaneus; head of the first, second, and fifth metatarsals; the lateral and medial epicondyle of the humerus; the styloid apophysis of the ulna and radius; and the second and fifth metacarpals (C-Motion, Germantown, MD, USA) [46]. These markers allowed for building a full-body biomechanical model in the Visual 3D software (Visual3D x64 Professional v6.01.36).

To collect the sEMG signal, the electrodes were placed over the muscle belly following the recommendations of the Surface ElectroMyoGraphy for the Non-Invasive Assessment of Muscles (SENIAM) [47] and the study by Sousa et al. [6] (Table 2). Electrode placement was confirmed by palpation. Before placing the electrodes, the skin was shaved, exfoliated to remove dead cells from the skin surface, and cleaned with isopropyl alcohol (70%) to remove oil and remaining dead cells. The electrode impedance checker was used to ensure that impedance levels were lower than 5 kΩ [47].

The collections were carried out in two moments, with a time interval of 72 h to 7 days [48,49,50].

#### 2.4.1. Task

Participants were asked to walk for 10 m, without technical aids, with their usual footwear, at a self-selected speed and without explicit instructions (“you can walk this route whenever you want”). Prior to data collection, sufficient time was given until the participants became familiar with the experimental setting [6,34].

The participants performed 20 valid trials: 10 trials with the DOM and IPSI limbs of the healthy and stroke group, respectively, in the LEAD position and with the NDOM and CONTRA limbs in the TRAIL position, and 10 trials with the opposite combination [49]. A resting time of two-minutes between trials was established to prevent fatigue [6,34]. The trials were considered valid if each lower limb, TRAIL and LEAD, made contact with each platform [34].

#### 2.4.2. Data Processing

Marker trajectories were processed through the Qualisys Track Manager software (Qualisys Track Manager 2020.3). Trajectory deviations or interruptions were interpolated using the linear, polynomial, and relational calculations built into the software. Subsequently, the resulting data were exported to the Visual 3D software, in which a full-body biomechanical model was built (according to the appropriate C-motion recommendations). Prior to exporting the data, a 6 Hz low-pass Butterworth filter was used for tracing the markers.

The hip, knee, and ankle joint position values in the sagittal plane were recorded during the heel strike of the LEAD limb and toe-off of the TRAIL limb. The heel strike was determined through the maximum horizontal distance between the marker of the ipsilateral calcaneus and the marker of the contralateral lateral malleolus [51,52]. The toe-off was determined through the minimum horizontal distance between the calcaneal marker and the sacral marker [52,53]. A positive variation in the range of movement refers to flexion for hip and knee joints and to plantar flexion for an ankle joint. The time of the double-support phase was calculated through the difference between the time of the heel strike and toe-off events.

The ground reaction force signal was used to calculate the WCOM of each lower limb during the double-support phase, assuming that the external mechanical power by a limb is equal to the dot product of the external force ($\vec{F}$) acting on the limb and the velocity of the centre of mass (${\vec{v}}_{com}$) [54]. Accordingly, in order to calculate the external work performed on the centre of mass, the velocity of the centre of mass (in the *F_y_* component) was first calculated through the first derivative of the centre of mass displacement. Then, the mechanical energy of the centre of mass for each limb (LEAD and TRAIL) was calculated by multiplying the respective ground reaction force (normalised by the mass of the participant) by the velocity of the centre of mass for the *F_y_* direction. Finally, the external work performed on the centre of mass for each member was computed by calculating the mechanical energy integral of each member in the double-support phase, according to Equations (2) and (3).

(2) $$WCOM_{trail\;=}{\vec{F}}_{trail\;}\times {\vec{v}}_{com\;=\;}F_{y,trail}v_{y,com}$$

(3) $$WCOM_{lead\;=}{\vec{F}}_{lead\;\;}\times {\vec{v}}_{com\;=\;}F_{y,lead}v_{y,com}$$

The electromyographic data processing was carried out using Matlab software, version 3.9.0. (MathWorks, Natick, MA, USA). A second-order digital band pass Butterworth with a cut of frequency between 20 and 450 Hz and the root mean square (RMS) value were calculated using a moving average window of 100 samples [25]. The mean of electromyographic activity during double support was normalised by the maximum value obtained during the gait cycle [34,55]. The coactivation ratio was calculated according to the following equation [56]:

(4) $$Coactivation\;ratio=\frac{\mathrm{agonist}\;\mathrm{activity}}{\mathrm{agonist}\;\mathrm{activity}+\mathrm{antagonist}\;\mathrm{activity}}$$

This coactivation ratio reflects a relative measure of agonist activity in a specific limb and in a specific position (LEAD or TRAIL) of the double-support phase [56]. The RF, VM, and TA muscles were considered the agonists for the LEAD position, while the GMax, BF, GasM, and SOL were considered the agonists in the TRAIL position. The coactivation ratio can vary between 0 and 1, with 0 indicating no agonist activity and 1 indicating no antagonist activity. In turn, a coactivation ratio of 0.5 indicates an agonist and antagonist coactivation in which their percentage of activation intensity was equal.

### 2.5. Statistical Analysis

IBM Statistical Package for the Social Science^®^ software version 28.0 (IBM Corporation, Armonk, NY, USA) was used for descriptive and inferential data analysis, with significance set at *p* < 0.05.

In order to ensure that there were no significant differences between groups (stroke vs. healthy) regarding age, mass, height, and BMI, the *t*-test for 2 independent samples was used. The assumption of normality was guaranteed using the Shapiro–Wilk test. For the comparison between groups regarding gender, Fisher’s test was used.

Mean and standard deviation were used as descriptive statistics for quantitative variables, and absolute and relative frequency for qualitative variables of sample characterisation (gender and injured/dominant side).

Intra-session and inter-session performance stability was verified by analysing the ICC. ICC values greater than 0.75 were considered good to excellent [32]. To determine the number of trials necessary to reach a good level of reliability (ICC > 0.75), the ICC values were calculated for each variable on the basis of n consecutive trials (n = 2 to n = 10 for inter-trial comparison and n = 1 to n = 10 for inter-session comparison). When the ICC did not reach values greater than 0.75 with 10 trials, the number of trials was classified as “>10”. For ICC processing, the absolute agreement was calculated for an average of measurements for all comparisons, except for the ICC referring to the comparison between sessions of only one repetition. In this case, an ICC of absolute agreement was used for a measurement [57].

A sample size calculator [58] was used to estimate the sample size [59,60].

## 3. Results

The number of trials required to achieve intra-session and inter-session ICC values higher than 0.75 for kinetic and kinematic variables are presented in Table 3. The number of trials needed in each session was generally low, ranging between two and three for most of the analysed variables, while between sessions, the values ranged from 1 to >10 trials. In some variables, more trials were required. In the TRAIL position, this was observed in the hip joint position in both limbs in both groups, as well as in the knee joint position in both limbs in the healthy group. In the LEAD position, this occurred in the hip joint position in both limbs in the stroke group, in the knee joint position in CONTRA, and in the ankle joint position in both limbs in the healthy group. These variables with low temporal consistency did not appear to show a clear pattern regarding differences between healthy and stroke groups nor limbs in each group. However, in the LEAD position, it can be observed that a higher number of trials for the hip position and a lower number for the ankle position were needed in the stroke group, contrasting with the opposite behaviour in the healthy group.

For double support time and the WCOM, the number of trials required ranged mostly between 1 and 3.

The number of trials required to achieve intra-session and inter-session ICC values higher than 0.75 for electromyographic variables is presented in Table 4. As in the kinematic variables, the number of trials required are in general lower intra-session (from 2 to >10) compared to inter-session (from 1 to >10) values. However, in both, the variability in most electromyographic variables seemed to be higher compared to the kinematics and kinetics. In many variables, 10 trials were not enough. These results indicate that when the CONTRA and the IPSI limbs were in the LEAD and TRAIL positions, respectively, a smaller number of trials was needed intra-session compared to the healthy group. When the limbs assumed opposite positions, the same did not occur, being verified, in most variables, with an increase in the number of trials in both groups. The muscles that needed a smaller number of trials (two) belonged to the stroke group.

It can also be observed that less trials seemed to be required in the CONTRA muscle activity between sessions compared to the healthy group.

## 4. Discussion

This study allowed us to determine the minimum number of trials needed to reach a good performance stability, both intra- and inter-session, of biomechanical variables reflecting interlimb coordination (kinematic, kinetic, and electromyographic) during the double support phase of gait in people with and without stroke sequelae. In post-stroke individuals, movement strategies seemed to be more stereotyped with less variability. This can be justified by the development of compensatory strategies, resulting from positional fixation [61,62]. Atypical muscle activation synergies and inadequate interarticular coordination can be suggested as the main causes for the development of these compensatory strategies.

### 4.1. Intra-Session

The results obtained in the present study revealed that intra-session, for most of the kinematic variables and for the kinetic variable studied, only two to three trials were enough to reach a good performance stability for an ICC > 0.75. The same did not happen in electromyographical variables, as a higher number of trials were required. Globally, the results of this study point to lower number of trials required in comparison with the ones suggested by previous studies. Fotiadou et al. [49] and Monaghan et al. [63] suggested that 10 trials seem to be enough to obtain excellent performance stability for most kinematic and kinetic variables of a gait cycle for the evaluation of both lower limbs, CONTRA and IPSI in gait, in people with stroke sequelae and healthy participants, respectively. The differences between the results of the present study and the previous one could be related to the fact that in this study, only the sagittal plane was considered and only the double-support phase. This motion plane seems to present a better performance stability compared to the frontal and transverse planes [49,64,65]. The results of joint kinematics in the sagittal plane, founded in our study, are in agreement with previous studies [49,64,65], demonstrating a low variability at a self-selected speed, which suggests that it may be acceptable to base clinical decisions according to the results of a single gait assessment.

The movement performance stability achieved with a lower number of trials for the joint position variables confirms that the movement variability is relatively stable when repeatedly performing a well-known automatic task [66], as is the example of walking at a comfortable self-selected speed. Kinematic variables can be considered spatial movement descriptors, independent of the forces that generate the movement [67]. Generally, it is assumed that gait kinematics is highly stable intra-session (repeatability) in healthy people [64], but also in people with stroke sequelae [65]. The intra-session variability seems to reflect the intrinsic (physiological) variability inherent in gait movement, such as gait velocity and pattern, as well as soft tissue movement [68], which being considered an automatic rhythmic movement is characterised by low variability [69].

The low number of intra-session trials for WCOM observed in the present study is in accordance with the work of Caty et al. [70] that highlighted the clinical importance of this variable. The variability of WCOM of the CONTRA limb of the stroke group in the TRAIL position seems to be under the evidence that the highest variability occurs in the limb that initiates the swing phase (TRAIL), and this could be explained by the instability produced in the transition of the lower limb from a closed kinetic chain to an open kinetic chain condition [49]. In the stroke group, when the CONTRA limb was in the LEAD position, less variability was observed compared to the healthy group in both limbs, and there was less variability of the CONTRA limb relative to IPSI in terms of lower limb muscular activity. This finding may point to a movement restriction causing a stereotyped pattern in the stroke group [49] when the CONTRA limb is in the LEAD position. On the other hand, the IPSI limb may have to make corrective adjustments in the face of the CONTRA movement pattern, thus increasing the variability of the IPSI [49] observed by the higher number of trials. The asymmetry between the two positions may be related to the specific role of each limb in each phase. In the LEAD position, the lower limb has a predominantly postural role for the initial contact of the foot with the ground and load response, while in the TRAIL position, the lower limb plays a propulsive role to initiate the swing phase [71]. A possible explanation could be related to the initial contact (LEAD), which in the CONTRA limb, being performed with less variability, translates into a more stereotyped movement strategy with less variability in muscle activation patterns, i.e., with a smaller repertoire of muscle activation patterns, and that could somehow influence the activity of the IPSI limb in the TRAIL position. Still, the higher number of repetitions in the CONTRA limb in the TRAIL position is consistent with the higher number of repetitions found in WCOM of that limb in the same position. A possible explanation for this result may be associated with the degree of hip extension in the TRAIL position, corresponding to the toe-off and early swing event, where the hip extension, by the eccentric action of the hip flexors [71], may be related to the propulsive function of this position. This hypothesis should be explored in future studies.

For electromyographic activity, the GMax in the CONTRA limb in both positions showed a lower variability, with only one to three trials being needed intra-session and inter-session. The lower variability of GMax may raise a hypothesis related to a low level of activity of this muscle, or to the search for alternative movement strategies to overcome the low level of activity, such as in more proximal segments, i.e., in the trunk, suggesting the inclusion of the analysis in future gait assessment studies. Globally, more than four trials are required when considering all muscles, and several muscles require more than ten. Although there are studies that have evaluated the repeatability of electromyographic variables in healthy people during gait [72,73], to the best of our knowledge, there are no studies on this type of assessment on post-stroke individuals.

Overall, although electromyographic activity is considered a gold standard in the assessment of muscle activity, the variability of electromyographic variables seems to be greater than the variability of kinematics and kinetics in both groups, healthy and stroke. The higher variability of electromyographic variables is aligned with the notion that various muscle activation strategies may give rise to similar kinematic patterns [74,75]. This higher variability over EMG might also be related with the highest difficulty in this assessment accuracy, justified by its random, non-stationary, and non-linear behaviour [76]. Trying to overcome this EMG feature, studies have already tried to develop better methodologies based on genetic algorithms [76] and the suitable number and position of EMG electrodes [77]. The number of trials required for these methods needs to be explored in future studies.

### 4.2. Inter-Session

Regarding inter-session variability (temporal consistency), the number of trials to reach a good performance stability ICC > 0.75 was higher compared to the intra-session, demonstrating greater variability between the two moments of analysis. Inter-session variability seems to reflect extrinsic variability parameters, associated with methodological errors, related to calibration or spatial resolution of motion capture systems, estimation of joint centres, marker application, marker movement on the skin in the case of kinematic and kinetic variables [65,68], and the placement of the electrodes and the normalisation process of the electromyographic signal. In the present study, in order to minimise methodological errors and their influence on the second data gathering, identical conditions were ensured, the same calibration method, and the replacement of the reflector markers and electromyographic sensors were applied by the same researcher/physiotherapist with experience in these assessment procedures.

The lower inter-session temporal consistency compared to the intra-session one observed in the healthy group, apart from methodological factors mentioned, may also have been due to an exploratory behaviour in the search for optimal movement solutions according to the task restrictions [78]. The results of this study in the healthy group demonstrated, in the LEAD position, a higher variability of the ankle joint position, compared to the other joints, that may have been related to joint variations in the initial contact for positioning the foot on the force plates. In the stroke group, the opposite was observed with less variable behaviour, which can be explained by the development of strategies to reduce degrees of freedom and increase stiffness in the ankle [79]. The lower variability in the ankle joint position may reflect the higher variability in proximal segments observed in the present study.

As observed intra-session, the double support time and WCOM seemed to also be more robust variables in both groups, healthy and stroke, inter-session. The decreased number of trials required for the IPSI limb of the stroke group in the LEAD position compared to the healthy group can result from a decreased capacity of the IPSI limb to adapt neuromuscular strategies to prepare the limb contact with the ground [6].

The search for reliable techniques to assess gait in people with stroke sequelae is crucial to define the design and monitoring of rehabilitation programs. Considering the clinical implications of the results of this study, for people with stroke sequelae, we can highlight the sagittal gait analysis with good-to-excellent repeatability in most kinematic and kinetic variables and its application for gait assessment in the chronic phase of post-stroke recovery. However, in the inter-session assessment, means should be sought to minimise the interference of methodological factors, thus allowing the feasibility of monitoring the intervention and evolution. A relevant implication for clinical practice may be the possibility of applying these measurements to develop individualised rehabilitation programs with person-centred clinical decisions [49]. The appropriate number of trials should consider the person under analysis and the parameter to be assessed, and therefore, Fotiadou et al. [49] suggested that the number of trials can be determined individually before each clinical assessment.

It would be interesting for future studies to investigate the repeatability of gait parameters in stroke patients with different gait speeds and different stages of motor recovery. Moreover, the identification of different parameters in gait behaviour, besides the laboratorial context, as explored in this study, is also important in terms of being understood in a real context. Some external characteristics of people (such as clothes or carried objects) [80] can be relevant to providing a better understanding or to identify gait movement patterns. Recently, some studies have already improved knowledge about human gait analysis [81,82], resorting to a computer vision. Some applications and techniques during gait feature assessment have already been suggested to predict and carefully analyse walking style in specific populations, such as individuals with Parkinson´s disease [82] and osteoarthritis [81]. It could also be interesting to apply this analysis on stroke sequelae individuals with concern for public safety in both indoor and outdoor contexts.

## 5. Conclusions

In this study, we were able to determine the minimum number of trials needed to reach good stability performance for kinematic, kinetic, and electromyographic variables reflecting interlimb coordination during the double-support phase in people with and without stroke sequelae.

The results of this study suggest that intra-session, for most of the kinematic variables in the sagittal plane (hip, knee, and ankle joint positions) and for the kinetic variable (WCOM), a low number of trials (2 to 3) seemed to be sufficient in order to achieve a good stability of the performance of the variables (ICC > 0.75), while in the electromyographic variables of the lower limb muscles, a higher number of trials seemed to be necessary (2 to >10). Inter-session, while one trial was needed for double support time, a higher number of trials seemed to be necessary for kinematic variables (1 to >10), for WCOM (1 to 9) and for electromyographic activity (1 to >10).

## Figures and Tables

**Table 1:** Participant characteristics (sociodemographic and clinical). Data presented from the mean (standard deviation (SD)). The *p*-value reflects the comparison between the stroke and healthy groups.

		Stroke (n = 11)	Healthy (n = 13)	Between-Groups Comparison
		Mean (SD)	Mean (SD)	*p*-Value
Age (years)		51.82 (12.92)	49.92 (14.91)	0.745
Weight (kg)		70.73 (11.15)	77.69 (15.87)	0.235
Height (m)		1.70 (0.13)	1.71 (0.12)	0.769
BMI (kg/m^2^)		24.58 (3.38)	26.29 (3.79)	0.261
Double-support time (s)				
	Ipsilesional/dominant	0.29 (0.31)	0.20 (0.03)	0.313
	Contralesional/non-dominant	0.24 (0.10)	0.19 (0.03)	0.162
Post-stroke time (months)		52.18 (29.89)	---	---

**Table 2:** Anatomical references for the electrode placement [34,47].

Muscle	Anatomical References
Tibialis anterior	On the third proximal of the line between the tip of the fibula and the tip of the medial malleolus
Soleus	Two centimetres distal to the lower border of the gastrocnemius medialis muscle belly and two centimetres medial to the posterior midline of the leg
Gastrocnemius medialis	Most prominent portion of the muscle belly
Rectus femoris	Fifty percent on the line between the anterior superior iliac spine and the upper border of the patella
Vastus medialis	Four centimetres above the superior chord of the patella and three centimetres measured medially and oriented 55 degrees from a reference line between the anterior superior iliac spine and the centre of the patella
Biceps femoris	Fifty percent on the line between the ischial tuberosity and the lateral epicondyle of the tibia
Gluteus maximus	Fifty percent on the line between the sacrum and the greater trochanter

**Table 3:** Number of trials needed to reach good performance stability intra-session and inter-session (ICC > 0.75) of the kinematic variables (hip, knee, and ankle joint position) and kinetics (work on the centre of mass) with the dominant lower limb (healthy)/ipsilesional (stroke) in the TRAIL and LEAD positions.

			Intra-Session				Inter-Session			
			Healthy		Stroke		Healthy		Stroke	
			DOM	NDOM	IPSI	CONTRA	DOM	NDOM	IPSI	CONTRA
Joint position										
	TRAIL									
		Hip	2	2	2	3	>10	>10	6	>10
		Knee	2	2	2	2	>10	5	2	3
		Ankle	2	2	3	2	1	2	4	2
	LEAD									
		Hip	2	2	2	2	2	2	>10	>10
		Knee	2	2	2	3	2	1	2	>10
		Ankle	2	4	2	2	5	>10	2	2
Time										
	Double support		2	2	2	2	1	1	1	1
WCOM										
	TRAIL		2	2	2	7	2	2	3	1
	LEAD		3	2	2	2	9	2	2	2

Number of trials to reach ICC > 75%; >10 (without reaching the goal). DOM: Dominant; NDOM: Non-Dominant; IPSI: Ipsilesional; CONTRA: Contralesional.

**Table 4:** Number of trials needed to reach good performance stability intra-session and inter-session (ICC > 0.75) of the electromyographic activity of muscles, tibialis anterior (TA), soleus (SOL), gastrocnemius medialis (GasM), rectus femoris (RF), biceps femoris (BF), vastus medialis (VM), and gluteus maximus (GMax) and the hip, knee, and ankle coactivation ratio with the dominant lower limb (healthy)/ipsilesional (STROKE) in the positions of TRAIL and LEAD.

			Intra-Session				Inter-Session			
			Healthy		Stroke		Healthy		Stroke	
			DOM	NDOM	IPSI	CONTRA	DOM	NDOM	IPSI	CONTRA
LEAD										
	Ankle									
		TA	>10	>10	10	7	>10	>10	>10	4
		SOL	8	7	7	2	2	>10	>10	5
		GasM	8	6	5	2	8	3	>10	>10
		Ankle ratio	7	4	7	2	5	2	>10	>10
	Knee and hip									
		RF	10	>10	7	7	>10	>10	>10	2
		VM	5	6	7	2	3	>10	5	>10
		BF	5	>10	4	8	6	>10	>10	>10
		GMax	7	>10	>10	3	>10	>10	>10	2
		Hip ratio	8	7	7	2	>10	>10	>10	1
		Knee ratio	8	>10	9	2	4	>10	>10	>10
TRAIL										
	Ankle									
		TA	7	3	10	5	1	>10	4	>10
		SOL	8	>10	2	7	4	>10	>10	8
		GasM	>10	>10	2	2	10	>10	1	>10
		Ankle ratio	5	7	2	>10	>10	7	>10	>10
	Knee and Hip									
		RF	9	7	2	8	>10	>10	>10	2
		VM	6	10	6	>10	2	>10	>10	>10
		BF	7	8	2	7	>10	>10	>10	>10
		GMax	6	6	>10	2	3	4	>10	1
		Hip ratio	5	7	3	2	>10	>10	>10	1
		Knee ratio	7	7	2	4	>10	>10	>10	>10

Number of trials to reach ICC>75%; >10 (without reaching the goal). DOM: Dominant; NDOM: Non-Dominant; IPSI: Ipsilesional; CONTRA: Contralesional.

## Data Availability

Not applicable.
